# Supplementary material for: Characterizing the Effects of Protein Glycosylation Perturbation on Phosphorylation Signaling
Source: bioRxiv. 2025 Dec 20:2025.12.18.695253. Preprint. [Version 1] doi: 10.64898/2025.12.18.695253 (PMC12724710; doi:10.64898/2025.12.18.695253)
Supplement: 1 [file NIHPP2025.12.18.695253V1-supplement-1.pdf]

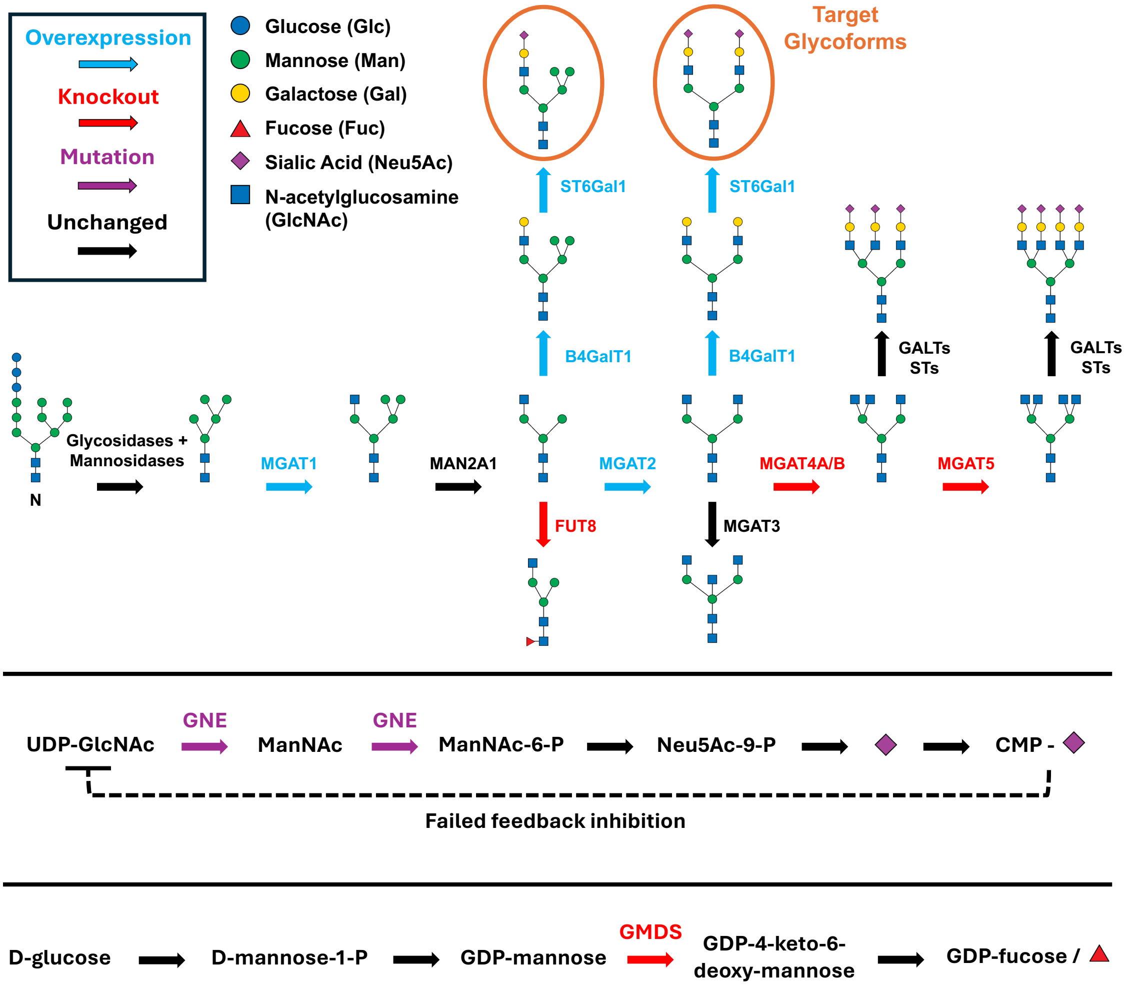

Supplementary Figure 1. Glycoengineering Schematic of this study in the ER-Golgi landscape. Genetic modifications are shown with colored arrows including overexpression, knockout, and mutation on the corresponding enzyme that catalyzes sugar addition; GALTs represent galatosyltransferases and STs represent sialyltransferases; circled glycoforms represent the engineered dominant target hybrid and target complex glycans. The GNE mutation for sialuria and GMDS knock-out schematic is also shown.

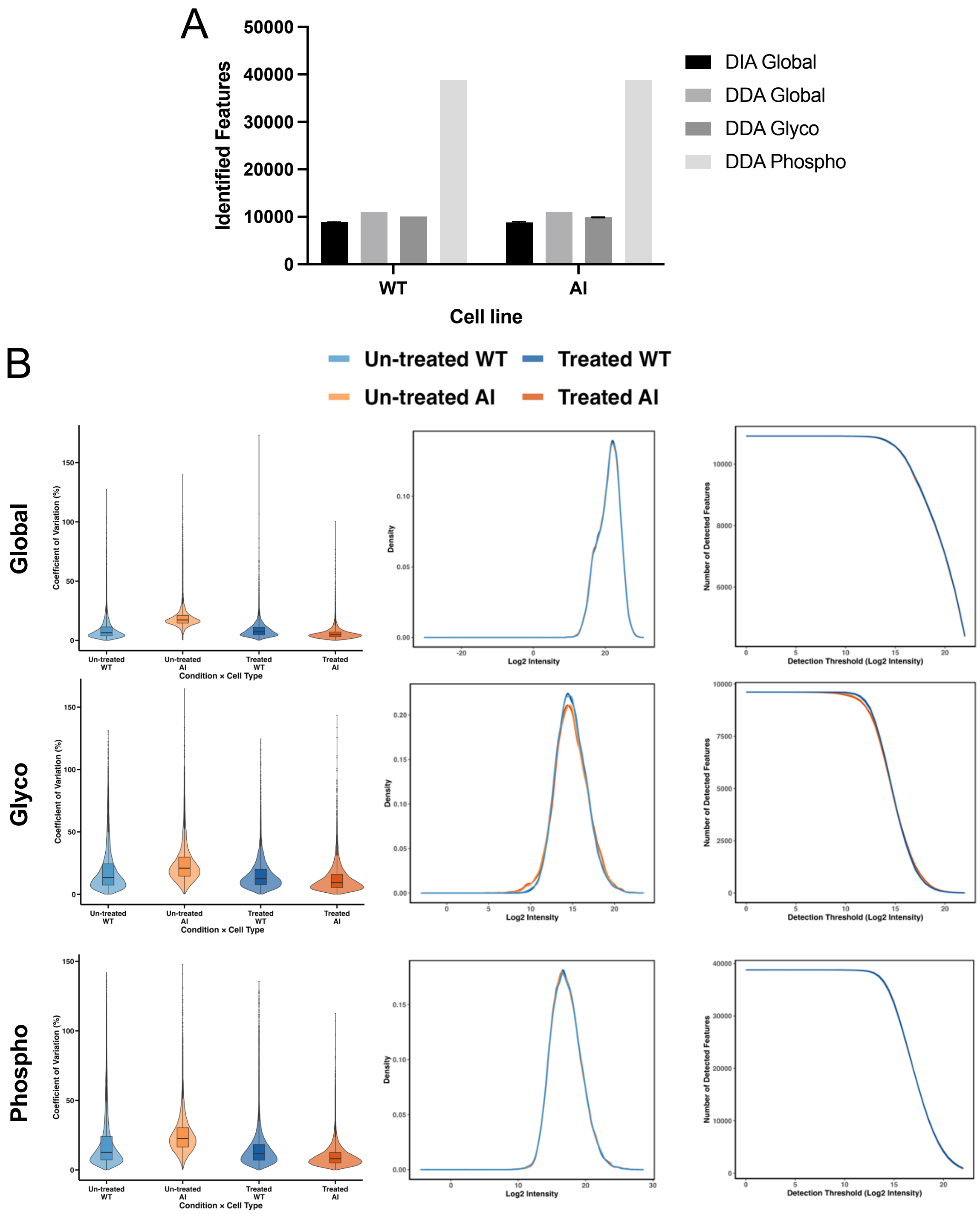

Supplementary Figure 2. A. Total identified features by reads in LC-MS/MS across both cell lines (WT, AI) and conditions (un-treated, treated) in DIA global, DDA global, glyco-, and phospho-datasets; some error bars are too small to be visualized due to the relative scale. **B.** Quality control for DDA global, glyco- and phospho-datasets including CV analysis, Gaussian kernel density estimation, and feature detection thresholds.

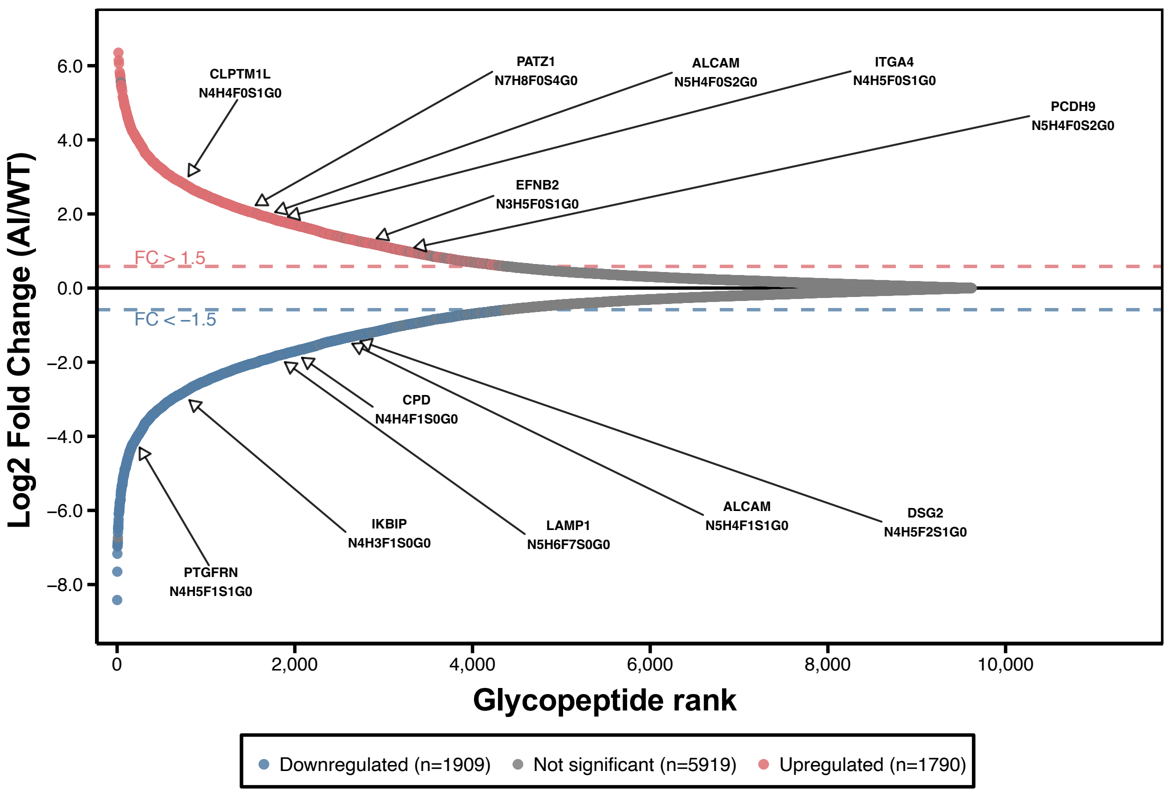

Supplementary Figure 3. S-plot of site-resolved glycopeptide analysis depicting regulation of various fucosylated, non-fucosylated, α2,6-sialylated, and non-sialylated glycopeptides labelled by corresponding gene.

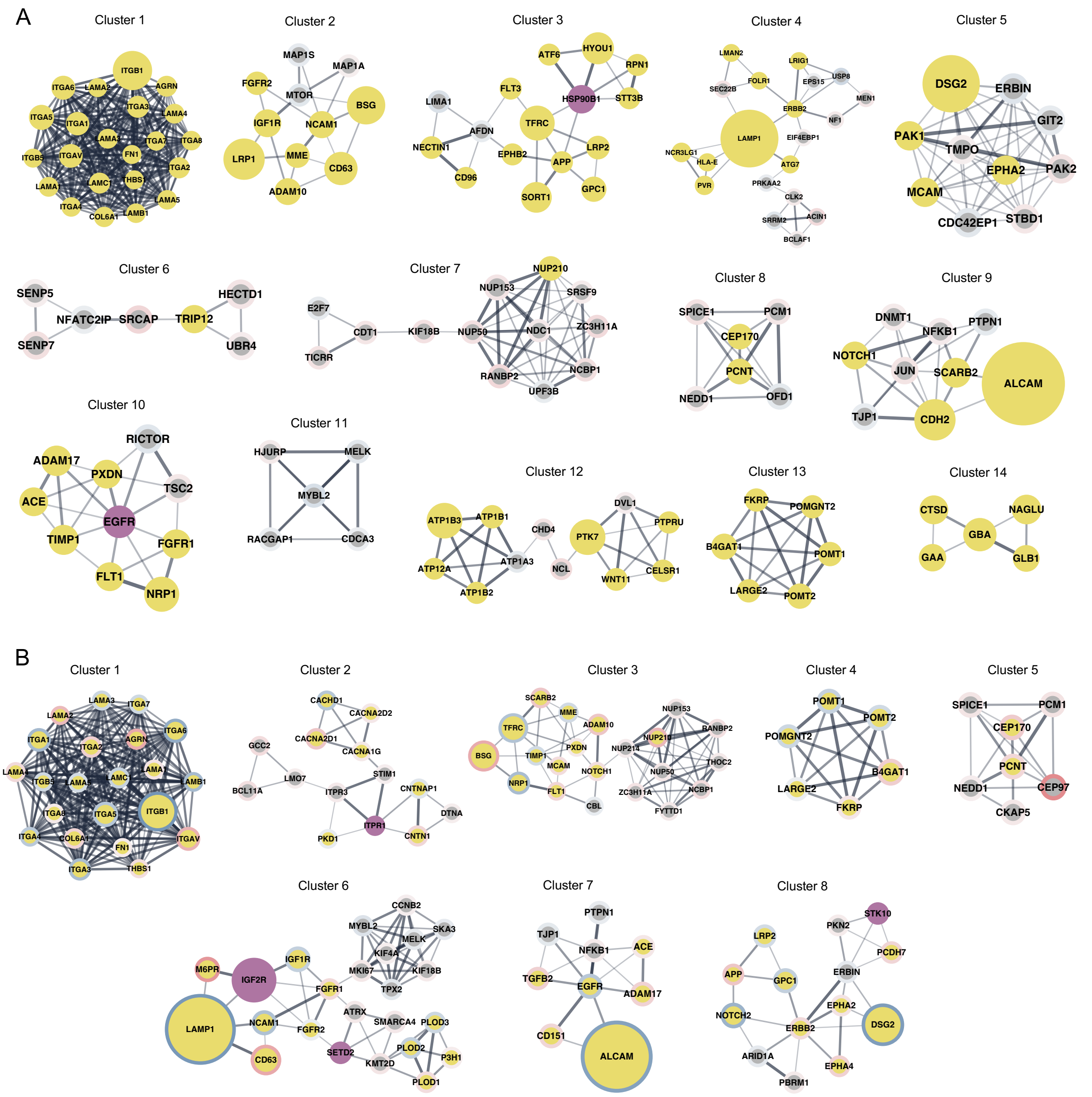

Supplementary Figure 4. All **A.** WT and **B.** AI MCODE cluster networks identified in Cytoscape. STRING Protein-Protein Interaction (PPI) extension of main Fig. 4 with similar legend correspondence, with the exception that node sizes are not to scale as in the main figure.
